# A review of phosphorus homeostasis and the impact of different types and amounts of dietary phosphate on metabolism and renal health in cats

**DOI:** 10.1111/jvim.15961

**Published:** 2020-11-06

**Authors:** Dottie Laflamme, Robert Backus, Scott Brown, Richard Butterwick, Gail Czarnecki‐Maulden, Jonathan Elliott, Andrea Fascetti, David Polzin

**Affiliations:** ^1^ Scientific Communications Consultant Floyd Virginia USA; ^2^ Department of Veterinary Medicine and Surgery College of Veterinary Medicine, University of Missouri Columbia Missouri USA; ^3^ College of Veterinary Medicine University of Georgia Athens Georgia USA; ^4^ Waltham Petcare Science Institute Leicestershire United Kingdom; ^5^ Nestlé Purina Research St Louis Missouri USA; ^6^ Royal Veterinary College University of London London United Kingdom; ^7^ Department of Molecular Biosciences University of California Davis California USA; ^8^ Veterinary Clinical Sciences Department College of Veterinary Medicine, University of Minnesota St Paul Minnesota USA

**Keywords:** calcium, homeostasis, kidney, magnesium, nutrition, phosphorus, toxicity

## Abstract

Elevated concentrations of serum phosphate are linked with progression and increased case fatality rate in animals and humans with chronic kidney disease. Elevated concentrations of serum phosphate can be a risk factor for development of renal and cardiovascular diseases or osteoporosis in previously healthy people. In rodents, an excess intake of dietary phosphorus combined with an inverse dietary calcium : phosphorus ratio (<1 : 1) contributes to renal calcification. Renal injury also has occured in cats fed experimental diets supplemented with highly soluble phosphate salts, especially in diets with inverse calcium : phosphorus ratios. However, not all phosphorus sources contribute similarly to this effect. This review, which focuses on cats, summarizes the published evidence regarding phosphorus metabolism and homeostasis, including the relative impact of different dietary phosphorus sources, and their impact on the kidneys. No data currently shows that commercial cat foods induce renal injury. However, some diets contain high amounts of phosphorus relative to recommendations and some have inverse Ca : P ratios and so could increase the risk for development of kidney disease. While limiting the use of highly soluble phosphates appears to be important, there are insufficient data to support a specific upper limit for phosphate intake. This review also proposes areas where additional research is needed in order to strengthen conclusions and recommendations regarding dietary phosphorus for cats.

**Abbreviations**
1,25D3: 1,25 dihydroxy vitamin D3
AAFCO: Association of American Feed Control Officials
Ca: calcium
CKD: chronic kidney disease
DM: dry matter
FEDIAF: Fédération européenne de l'industrie des aliments pour animaux familiers
FGF23: fibroblast growth factor 23
GFR: glomerular filtration rate
Mg: magnesium
NRC: National Research Council (USA)
P: phosphorus
Pi: phosphate
PTH: parathyroid hormone
SDHP: sodium dihydrogen phosphate
sPi: serum phosphate concentration
uPi: urinary phosphate

## 1 INTRODUCTION

Phosphorus (P) is a nutritionally essential macromineral and is involved in most, if not all, of the metabolic reactions in the body, including energy metabolism, bone and teeth formation, osmotic and acid‐base balance, electrolyte transport, and numerous enzyme systems; and is a constituent of nucleic acids.^1^, ^2^, ^3^, ^4^ Most nonskeletal P in the body is intracellular. The body controls extracellular P concentrations primarily by regulation of urinary excretion but also by intestinal absorption and deposition into and release from bound sources in bone and other tissues.

Although P is essential, excess can be detrimental. Elevated concentrations of blood phosphate or maladaptive activation of hormonal systems involved in regulating phosphate could have direct toxic effects on the kidneys and cardiovascular system.^5^, ^6^ Reducing serum phosphate concentration (sPi) in subjects with chronic kidney disease (CKD) delays progression and death.^7^, ^8^, ^9^, ^10^, ^11^, ^12^ Evidence in humans suggests that elevated sPi (in the high normal range or highest quartile in population studies) can be a risk factor for development of renal disease, cardiovascular disease, or osteoporosis, and is associated with increased mortality.^13^, ^14^, ^15^, ^16^ Although there are discrepancies between dietary P and sPi, dietary factors do contribute to sPi, as detailed below. Human diets considered to increase risk for elevated sPi are those high in added phosphates (Pi) and low in calcium (Ca), resulting in inverse Ca : P ratios (phosphorus greater than calcium) which is itself a risk factor for renal injury.^17^, ^18^ In cats, under some circumstances, intake of highly soluble Pi salts can induce damage to otherwise healthy kidneys.^19^, ^20^ The detrimental effects of the Pi salts are not limited to clearly excessive dietary P nor inverse dietary Ca : P ratios. In some cases, the effects occur during feeding of diets containing total P concentrations not in excess of that found in some commercial diets, although the sources and proportions of added Pi in the experimental diets differ from those in commercial diets.

This review summarizes the published evidence regarding P metabolism and homeostasis, including how these factors affect the kidneys. This review further notes the limitations and gaps in the existing evidence, and proposes areas where additional research is needed in order to strengthen conclusions and recommendations regarding dietary P for cats.

## 2 PHOSPHORUS DIGESTION, METABOLISM, AND HOMEOSTASIS IN MAMMALS

Dietary P is absorbed in the small intestine via 2 general mechanisms: sodium‐dependent active absorption (primary route), and concentration dependent paracellular diffusion (secondary route) in humans and rodents. Active absorption of P is up‐ or down‐regulated depending on the animal's needs and various regulating factors, while paracellular diffusion is more dependent on the total amount consumed.^21^, ^22^, ^23^ Parathyroid hormone (PTH), 1‐25‐dihydroxy vitamin D3 (1,25D3), fibroblast growth factor 23 (FGF23), thyroid hormone, glucocorticoids, estrogens and metabolic acidosis are just some of the endogenous metabolic factors that can modify intestinal P uptake either directly or indirectly.^2^, ^23^, ^24^, ^25^ In addition, P absorption in most species is dependent on the intestinal pH, P needs of the animal, source of P, and interactions with other dietary factors such as dietary Ca, magnesium (Mg), and phytates.^2^, ^21^, ^24^, ^25^, ^26^, ^27^, ^28^, ^29^, ^30^, ^31^ Metabolic acidosis upregulates sodium‐dependent P uptake in the intestines of mice while simultaneously increasing urinary excretion of phosphate (Pi).^2^ Dietary Ca and Ca : P ratio exert an influence over P availability, with intestinal absorption of P inversely affected by dietary Ca and Ca : P ratio, thus increasing when Ca : P is low. This has been demonstrated in dogs and cats, as well as other animals.^21^, ^32^ Likewise, dietary Mg affects intestinal uptake of both Ca and P in both rats and cats, with higher Mg decreasing uptake of Ca and P.^26^, ^31^, ^33^, ^34^

Increased dietary protein appeared to enhance renal Pi excretion and decrease Pi retention in both adult and growing cats, so P requirements might be dependent on dietary protein content.^28^, ^35^, ^36^ This effect could be an artifact of protein's effect on pH. Since Pi is a critical component of urinary titratable acids used to remove excess acid from the body in a buffered form (dihydrogen phosphate), an acidic pH can increase urinary Pi (uPi) excretion. This has been confirmed in cats in 1 study designed to evaluate dietary base excess on urine pH.^37^ In that study, there was a significant, negative correlation between urinary pH and uPi. Similarly, in cats given phosphoric acid to increase urinary acidification, average 24‐hour Pi excretion was significantly higher than in cats with less acidic urine despite identical dietary P concentrations.^38^ Thus, as urine becomes less acidic, uPi decreases which could increase positive P balance and increase sPi.

Dietary P can be intrinsic to ingredients, such as meats, bone meals, and grains, or be added as inorganic sources such as mined phosphate rock, phosphoric acid, or various phosphate salts. (Table 1). Phosphorus from animal sources is primarily present as calcium phosphates (such as hydroxyapatite) and phosphate esters. The apparent digestibility of P ranges between 0% and 80% depending on source and other dietary variables. Grain and oilseed based P is poorly digested because it is primarily present as phytate, with digestibility between 0% and 40%.^16^, ^22^, ^39^ Supplemental inorganic Pi, such as those in phosphate‐containing food additives, have an intestinal uptake of 80% or greater.^16^, ^39^ One study comparing cats fed P coming primarily from poultry, meat, fish meals with added inorganic Pi (mono‐ and di‐basic sodium phosphates) confirmed greater intestinal uptake of the P from sodium phosphates, with about 40% absorption compared to about 20% absorption for the diet containing primarily animal sources of P.^27^ Concurrent with this were increases in sPi and uPi in cats fed the more digestible sodium phosphate containing diet.^27^ Although supplemental inorganic P sources are generally more bioavailable than those intrinsic to food ingredients, they are not uniform is this respect. In dogs, monosodium phosphate (NaH_2_PO_4_) and monopotassium phosphate (KH_2_PO_4_) significantly increased sPi and PTH compared to similar amounts of P from poultry meal (Herbst S, Dobenecker B. Effects of phosphorus addition from organic and inorganic sources on kinetics of selected blood parameters in dogs. Proc Eur Soc Vet Clin Nutr 2018, Munich, Germany. Abstract) while another study showed that the effects of calcium diphosphate (Ca[H_2_PO_4_]_2_) were intermediate between phosphorus from poultry meal and monosodium phosphate (Siedler S, Dobenecker B. Effect of different P sources in high phosphorus diets with balanced Ca/P ratio on serum PTH, phosphorus and calcium levels as well as apparent digestibility of these minerals in dogs. Proc Eur Soc Vet Clin Nutr 2015, Toulouse, France. Abstract).

**TABLE 1:** Names and chemical formulas for phosphate salts

Chemical formula^a^	Common name^a^	Alternate names^a^	Used in research cited in this document	Used in cat foods (type)^b^
NaH_2_PO_2_	Sodium hypophosphite			
Multiple	Sodium phosphate	Used as generic term for mono‐, di‐, and tri‐sodium phosphates		Wet
NaH_2_PO_4_	Monosodium phosphate	Sodium Dihydrogen phosphate, SDHP; Sodium phosphate monobasic, sodium dihydrogen orthophosphate	Dobenecker 2017‐cats; Dobenecker 2018‐cats; Alexander 2018‐cats; Matsuzaki 1999‐rats; Finco 1999‐cats	Dry, wet
Na_2_HPO_4_	Disodium phosphate	Disodium hydrogen phosphate, sodium phosphate dibasic	Coltherd 2018‐cats; Finco 1999‐cats	Dry, wet
Na_3_PO_4_	Trisodium phosphate	Sodium orthophosphate, tribasic sodium phosphate; trisodium orthophosphate, TSP		Wet
Na_5_P_3_O_10_	Sodium tripolyphosphate^c^	Pentasodium triphosphate, STPP	Coltherd 2018‐cats; Matsuzaki 1999‐rats	Dry, wet
Na_4_P_2_O_7_	Tetrasodium pyrophosphate	TSPP, Sodium pyrophosphate		Dry, wet
(NaPO_3_)_6_	Sodium hexametaphosphate	Sodium polymetaphosphate		Dry, wet
H_3_PO_4_	Phosphoric acid^d^	Orthophosphoric acid		Dry, wet
CaH_4_(PO_4_)_2_; OR Ca(H₂PO₄)₂	Monocalcium phosphate	Calcium biphosphate, Calcium monobasic phosphate, Calcium dihydrogen phosphate	Dobenecker 2017‐cats; Dobenecker 2018‐cats	Dry, wet
Ca_3_(PO_4_)_2_	Calcium phosphate	Tricalcium orthophosphate, penta‐Calcium hydroxide triphosphate, Calcium phosphate tribasic		Dry, wet
CaHPO_4_	Dicalcium phosphate^c^ ^,^ ^d^	Calcium hydrogen phosphate, calcium phosphate dibasic	Cockell 2002 and 2004‐rats; PaBlack 2016‐cats;	Dry, wet
Ca_3_(PO_4_)_2_	Tricalcium phosphate^c^	Calcium phosphate		Dry, wet
Ca_10_(PO_4_)_6_(OH)_2_	Calcium apatite	Hydroxyapatite		
Ca_4_Na(PO_4_)_3_	Defluorinated phosphate	Defluorinated feed phosphate		Dry, wet
KH_2_PO_4_	Monopotassium phosphate	Potassium dihydrogen phosphate	Cockell 2002 and 2004‐rats; Tani 2007‐rats; Matsuzaki 1999‐rats	Dry, wet
K_2_HPO_4_	Dipotassium phosphate	Potassium phosphate dibasic		
K_3_PO_4_	Tripotassium phosphate	Potassium phosphate, potassium phosphate tribasic		
K_5_P_3_O_10_	Potassium tripolyphosphate	Pentapotassium triphosphate, potassium triphosphate	Matsuzaki 1999‐rats	
K_4_P_2_O_7_	Potassium pyrophosphate	Potassium diphosphate, tetrapotassium diphosphate; tetrapotassium pyrophosphate		

^a^ Primary resource for chemical names and formulas: Pub Chem: US National Library of Medicine.

^b^ US Cat foods, based on ingredient information listed on Chewy.com, accessed February 2019, and Mintel's Global New Products Database (gnpd.com) accessed February 2020.

^c^ Among most common phosphate additives used in wet cat foods in USA (gnpd.com).

^d^ Among most common phosphate additives used in dry cat foods in USA (gnpd.com).

Meta‐analysis of digestion trials in dogs and cats estimated the average true digestibility of P to be 17% in dogs and 31% in cats.^32^ In both dogs and cats, as in other species, Ca : P ratio affects P digestion : higher ratios are associated with lower digestibility. Adult cats fed diets with Ca : P ratios of 1 : 1 have apparent digestibility of P averages about 50%, while increasing Ca to a ratio of 2 : 1 or 4 : 1 reduces apparent absorption.^40^ The reverse also holds true, with P digestibility increased when the Ca : P ratio is less than 1 : 1.^30^, ^32^ The average true digestibility of dietary P was summarized as: 49% when the Ca : P was less than 1 : 1; 27% when the Ca : P ratio fell between 1 : 1 and 2 : 1; and approximately 0% when Ca : P was greater than 2 : 1.^32^ It is not known how much of this effect is strictly the Ca : P ratio or if differences in P sources or other dietary variables affect the apparent digestibility of P.

Dietary Mg is another macromineral that interacts with Ca and P. In cats, as dietary Mg (added as MgCO_3_) increases, intestinal absorption of dietary P and urinary excretion of Pi are both reduced in a linear manner.^31^ In addition to impact on Pi homeostasis, low serum Mg is correlated with CKD, cardiovascular disease and all‐cause mortality.^6^, ^41^, ^42^ Mg can reduce vascular mineralization in the face of hyperphosphatemia, reduce sPi, and reduce FGF23 which independently contribute to death in CKD.^6^, ^41^, ^42^, ^43^

In healthy subjects, nearly 100% of sPi is filtered via the renal glomerulus and 80% to 90% is typically reabsorbed via sodium‐mediated facilitated cotransporters in the renal tubules.^16^ That portion not reabsorbed is excreted in the urine. Total renal phosphorus excretion is balanced to phosphorus intake under the control of PTH, 1,25D3, and FGF23 (and its cofactor α‐klotho) so as to maintain sPi within a fairly stable range.^4^, ^44^ In cats, for example, acute responses to high intake of readily available inorganic phosphate appear to involve rapid secretion of PTH, resulting in increased uPi.^45^ Adaptation to longer term feeding of cats with diets high in readily available phosphate results in increases in both FGF23 and PTH, both increasing uPi.^19^ In a healthy adult animal at steady state (not pregnant or lactating), the total urinary Pi is thought to approximate the amount absorbed in the intestines,^46^, ^47^ although exceptions to this have been noted.^47^, ^48^ In particular, with inadequate intake of P or low absorption of P from the intestine, uPi is low and may serve as an indicator of altered P regulation.^44^, ^49^ In the face of kidney disease, however, urinary excretion of P is compromised.^50^

The amount of phosphorus entering renal tubules is dependent on glomerular filtration, as there are no other mechanisms by which additional Pi can be added to the filtrate. Thus, for a given sPi, the amount of Pi entering the tubules is dependent on glomerular filtration rate (GFR). As GFR decreases in the face of CKD, the amount of filtrate decreases. Likewise, for a given GFR, the sPi affects the amount of Pi entering the tubules. Thus, as sPi increases, the amount of Pi entering the tubules increases despite the reduced GFR. Once in the renal tubules, reabsorption or excretion is controlled by PTH, 1,25D3, and FGF23, as noted above. However, these also are impacted by CKD. Both FGF23 and PTH are increased in CKD which result in reduced renal tubular reabsorption of Pi and greater urinary Pi excretion, reduced activation of 1,25D3 and reduced intestinal P absorption,^51^maintaining stable sPi despite reduced GFR. As CKD progresses, however, there is a reduction in kidney‐derived α‐klotho, an essential cofactor for FGF23.^25^, ^52^ In the absence of α‐klotho, FGF23 is nonfunctional, thereby limiting the kidney's ability to regulate Pi reabsorption despite increased concentrations of FGF23. As the ability to regulate Pi homeostasis continues to decline with advancing CKD, sPi increases until a new equilibrium is reached but at a greater sPi. The increase in PTH, FGF23 and sPi that occur in CKD all are associated with ongoing renal damage and increased mortality rates.^53^, ^54^, ^55^

## 3 PHOSPHORUS DYSREGULATION AND IMPACT ON RENAL HEALTH

Hyperphosphatemia contributes to ongoing damage in the face of existing kidney disease, and dietary P restriction can help reduce this ongoing damage.^8^, ^16^, ^56^, ^57^, ^58^ In humans, hyperphosphatemia secondary to CKD is linked with disease progression, increased vascular damage and cardiovascular morbidity and death.^16^, ^23^, ^58^ In mice with CKD fed high P diets (ie, 0.9% P vs 0.5% P; Ca : P 0.66 : 1 vs 1.2 : 1), high P intake results in vascular calcification which can aggravate CKD or contribute to cardiovascular disease.^59^, ^60^ Elevations in serum PTH and FGF23, which are stimulated in healthy subjects by increased P intake and which are increased in patients with established CKD, are associated with increased risk of CKD progression^6^, ^61^ Likewise, in cats, elevations in sPi, PTH and FGF23 are risk or prognostic factors for all‐cause mortality and progression of CKD.^55^, ^62^, ^63^, ^64^, ^65^ Serum Pi is positively correlated with severity of interstitial fibrosis in cats with CKD.^66^ However, it is not known if these substances actually contribute to renal injury, or are simply markers of ongoing renal injury.^16^

Female rats are predisposed to development of nephrocalcinosis, or calcification of kidney tissue especially at the corticomedullary junctions. This form of kidney disease is responsive to dietary P and to the dietary Ca : P ratio.^18^, ^67^, ^68^, ^69^, ^70^ An inverse Ca : P ratio (Ca less than P) appears to be the primary risk factor although absolute amounts of Ca and P also influence nephrocalcinosis.^18^, ^68^ For example, when female rats were fed a diet that contained approximately 5 g Ca and 6 g P/kg dry diet and had a molar Ca : P ratio of about 0.66 : 1, kidney tissue accumulated 171 ± 130 μmol calcium/g dry tissue weight. In the same study, female rats fed diets modified with considerably more Ca and P but molar Ca : P ratios of 1.1‐1.2 : 1 showed significantly less calcification. The highest Ca diet, containing 20 g Ca and 13 g P/kg dry diet, with a molar Ca : P ratio of 1.2 : 1, resulted in only 39 ± 31 μmol calcium/g dry tissue weight despite containing 4× more Ca and 2× more P than the diet with the inverse Ca : P ratio.^68^

In dogs with induced CKD, restriction of dietary P reduces the associated morbidity and improves survival.^56^, ^71^, ^72^ In cats with induced renal failure, restriction of dietary P from 1.56% of diet dry matter (DM) to 0.42% results in less renal mineralization, fibrosis and inflammatory cell infiltration.^7^ Cats on the lower P intake also have lower serum Pi and PTH concentrations compared to those fed the high P diet. It is important to note that the high P diet used a highly soluble inorganic P salt mixture (Na_3_PO_4_) for a large portion of the P (about 73% of total P, or 1.1% diet DM added Pi) and also had an inverse Ca : P ratio, both of which can alter bioavailability and renal effects of P.^7^ While no studies have evaluated P restriction alone in cats with naturally occurring CKD, several do show clinical benefits in cats with CKD fed “renal diets” (restricted in phosphorus, protein, and sodium, plus alkalinizing buffers and often with other changes compared to maintenance diets) or those treated with phosphate binders.^9^, ^10^, ^73^, ^74^

Evidence in people has been accumulating that elevations in sPi can be a risk factor for development of renal disease, cardiovascular disease and increased mortality even in patients with apparently normal kidney function.^14^, ^15^, ^16^, ^17^ These epidemiological studies evaluated sPi rather than intake, and the association between dietary P and sPi is weak at best.^58^, ^75^, ^76^ Discrepancies between dietary P and sPi in healthy subjects are due to several factors including normal diurnal variation in sPi and efficient excretion of excess P such that fasting sPi does not change with alterations in P intake.^15^, ^77^, ^78^Serum Pi, which represents only about 1% of total body P, also can be affected by fluxes into and out of intestine, bone and intracellular spaces.^76^, ^78^ Additional factors include differences in bioavailability of various P sources, and other dietary factors that can alter P absorption, such as the dietary Ca : P ratio.^15^, ^45^, ^58^ Studies evaluating P intake yielded mixed results regarding impact on morbidity or mortality rates. For example, the NHANES III study found no association between dietary P and mortality in patients with compromised renal function, yet another study in dialysis patients reports a strong association between dietary P and mortality rate.^16^ In the NHANES III study, analysis of data from the subset of subjects that were healthy at the start of the study demonstrated a positive association between diet P and mortality among individuals consuming more than 1400 mg per day.^79^ However, in that same study, diets with the lowest P concentrations (energy basis) were also associated with greater all‐cause mortality.^79^ A study in human patients with or without diabetes mellitus documented an increased risk for development of CKD in those diabetic patients consuming the highest P diets, but no impact was observed in the nondiabetic subjects.^80^

A small study in pet dogs and cats suggests a positive correlation between high P intake (assessed through dietary questionnaire) and CKD in cats, but not in dogs.^81^ Median P intake at the time of diagnosis was 239% of the recommended allowance in cats with CKD and 147% of the recommended allowance in the controls. Home‐made diets were the most commonly fed diet type in this study, and the P sources and Ca : P ratio were not reported. In a different study of aged cats (10 years of age or older) without clinical evidence of CKD, a higher P diet (2.6 g/Mcal) had no impact on development of azotemic CKD compared with those fed a lower P diet (1.6 g/Mcal) over an 18‐month period.^77^ It should be noted that in this study the overall prevalence of azotemic‐CKD was less than expected, so the study might have been underpowered to detect an effect from P intake.

High Pi concentrations alter vascular and endothelial function and alter the nitric oxide pathway in in vitro studies.^82^, ^83^ It is hypothesized that these changes can contribute to increased proteinuria, which might contribute to further renal injury.^16^ In human patients with and without CKD, there is a significant association between sPi or uPi excretion and proteinuria.^16^ The endothelial effects have been confirmed with in vivo studies in humans and rats.^82^, ^83^, ^84^, ^85^ For example, healthy men fed 1200 mg P (vs 400 mg P) show 2‐hours postprandial elevations in sPi and compromised vascular function measured as flow‐mediated dilation of the brachial artery. However, the Ca : P ratio in this treatment group was 0.167 : 1.^82^ The lower the dietary Ca : P ratio, the higher the apparent likelihood of adverse effects related to any given dietary P intake.^58^ Lack of control of the Ca : P ratio is a common confounding issue in studies investigating the effects of high P intake.^15^, ^16^, ^70^, ^79^, ^82^, ^83^, ^84^

## 4 EVIDENCE FOR PHOSPHORUS' ROLE IN RENAL HEALTH IN CATS

Although there is currently no evidence that P in commercial cat foods induces renal disease in healthy cats, high “ash” content in food might be a risk factor for developing CKD.^86^ Ash refers to all the minerals in food, and this includes P but is not specifically correlated to it. In that study, higher intakes of dietary fiber, magnesium and sodium all were associated with reduced risk for CKD. Each of these nutrients can impact dietary P availability. Another small study suggests that increased P and protein intakes might be associated with CKD in cats.^81^

Studies in healthy cats show an impact of dietary P, or inverse Ca : P ratios, on markers of kidney function.^19^, ^20^, ^87^, ^88^ In the earliest study, cats were fed 4 purified diets each for 28 days with incremental increases in dietary P (4.6, 9.2, 18.4, and 27.7 mmol/MJ [0.28%, 0.56%, 1.12%, and 1.71% of dry diet]). Dietary Ca was held constant so that the Ca : P ratio decreased with increasing dietary P, provided as sodium dihydrogen phosphate dihydrate (NaH_2_PO_4_‐2H_2_O). The Ca : P ratios ranged from 1.8 : 1 down to 0.3 : 1.^87^ At the highest P level tested (with lowest Ca : P ratio), food intake decreased; sPi was reduced and serum alkaline phosphatase increased. Also, in this diet group, creatinine clearance decreased, suggesting an adverse impact on renal function in otherwise healthy cats. Fasting sPi was reduced with the higher dietary P and reduced GFR, although the significance of this is not known.

In the second study, healthy cats developed glucosuria, microalbuminuria and showed significantly decreased creatinine clearance within 28 days of being fed a diet containing approximately 3 g P/Mcal ME (1.6% of diet DM) with approximately 2/3 provided as a combination of monocalcium (CaH_4_[PO_4_]_2_) and monosodium‐(NaH_2_PO_4_) phosphates.^20^ The amount of phosphorus from highly soluble monosodium phosphate was approximately 0.4 g/Mcal ME or 0.17% of diet DM, in a diet with a Ca : P ratio of approximately 0.4 : 1.

A third study compared a diet supplemented with sodium dihydrogen phosphate (SDHP: NaH_2_PO_4_, also referred to as monosodium phosphate) to deliver 4.78 g P/Mcal ME (1.93% diet DM) with a Ca : P ratio of 0.6 to a control diet providing 1.23 g P/Mcal ME (0.52% diet DM) and Ca : P at 1.1.^19^ Within 4 weeks after starting on this HP‐NaP diet, cats developed changes consistent with renal injury: increased plasma creatinine and urea nitrogen; increased urinary microalbuminuria; decreased GFR; and ultrasound echogenicity changes consistent with altered renal morphology. GFR also decreased in the control group, but to a lesser extent than the HP‐NaP group. A final study evaluated a more moderate diet providing 3.6 g P/Mcal ME (1.5% diet DM), including approximately 1.5 g P (0.6% diet DM) from SDHP, and a Ca : P ratio of 0.9.^19^ Urinary albumin excretion was significantly increased in the higher P cats by week 4 and remained elevated throughout the study. Three cats (12.5%) fed this diet developed biochemical evidence of kidney disease and 36% of the cats showed evidence of altered renal morphology. In addition, 15 cats (60%) fed this diet developed renal stones, compared with 6 cats (27%) fed the control diet.^19^ Thus, multiple studies show adverse renal effects in cats fed diets containing highly soluble inorganic Pi especially, but not exclusively, in diets with a Ca : P ratio less than 1 : 1—findings consistent with those reported in other species. A key question not addressed in these studies is the applicability of these findings to other P sources.

The studies above did not compare the effects of different P sources on renal biomarkers, however other research evaluated different P sources on other physiologically important parameters. One study compared a mix of dietary P sources (poultry, meat, fish meals, and calcium phosphate) with supplemental inorganic P from mono‐ and di‐basic sodium phosphates, and confirmed significantly greater uptake, along with increases in sPi and uPi in cats fed the more digestible inorganic P.^27^ Two studies in cats compared diets providing P only from rice and beef (total P approximately 0.54%‐0.64% DM) with high P diets providing additional P from either monosodium phosphate (HP‐NaP: total P, 1.7% DM) or monocalcium phosphate (HP‐CaP: total P, 1.5% DM).^88^ Monosodium phosphate is a water soluble P source whereas monocalcium phosphate is poorly water soluble.^89^ The Ca : P ratio was held constant for all diets at about 1.3 : 1. Each diet was fed for 29 days. Urine volume doubled in cats fed HP‐NaP diet (which was high in sodium as well as P). Despite this increase in volume, uPi concentration increased. Glucosuria occurred in 54% of cats fed this diet but did not occur with the HP‐CaP diet. The authors concluded the differences in phosphaturic effects were due to the high solubility of monosodium phosphate.^88^

A different study design was used in series of acute kinetic trials in cats fed diets with 2 different inorganic sodium phosphate sources, SDHP and sodium tripolyphosphate (Na_5_P_3_O_10_).^45^ Each randomized, cross‐over trial measured relevant blood parameters (plasma Pi, PTH and FGF‐23 concentrations, and whole blood ionized Ca) over a 6‐hour postprandial period with no dietary adaptation period. The trials indicate a dose‐dependent and threshold effect from the supplemental sodium phosphates on plasma Pi: at 0.5 g P/Mcal (0.64 g kg diet DM) or greater added inorganic Pi, plasma Pi and PTH increases and blood iCa decreases, whereas with lesser additions or with increased food‐source P, no increases in plasma Pi or PTH occurs during the 6‐hour postprandial period. Furthermore, the Ca : P ratio of the diet affects the response to dietary sodium phosphates, with a prolonged elevation in plasma Pi in cats fed diets with Ca : P less than 1 : 1.^45^ Given that prolonged elevations in blood Pi are associated with renal injury and increased all‐cause mortality, at least in humans, this can have clinical importance.^14^

## 5 PHOSPHORUS IN COMMERCIAL CAT FOODS

Phosphorus sources have many uses in pet food in addition to their contribution to nutritional adequacy. Some of these functional uses include: control of urine pH for prevention of stone formation (ie, phosphoric acid); reduced accumulation of dental tartar (ie, sodium hexametaphosphate); and, processing aids to ensure optimal cooking and texture (ie, sodium phosphates). Among commercial cat foods in the United States, the most commonly added phosphorus sources for wet foods include tricalcium phosphate, dicalcium phosphate, and sodium tripolyphosphate, while phosphoric acid and dicalcium phosphate are the most commonly added sources of phosphate in dry cat foods (Table 1).

United States pet food regulations address both nutrient content of foods and ingredients used. Table 2 lists the recommendations for Ca and P levels as cited by the NRC (National Research Council), AAFCO (Association of American Feed Control Officials), and FEDIAF (Fédération européenne de l'industrie des aliments pour animaux familiers—The European Pet Food Industry Federation).^90^, ^91^, ^92^ None of these organizations currently list a maximum or safe upper limit for phosphorus for cat foods although FEDIAF does list a maximum for the Ca : P ratio of 2 : 1, and both FEDIAF and AAFCO provide maximum limits for phosphorus for dog foods. In 2019, FEDIAF added a footnote relative to P, stating “High intake of inorganic phosphorus compounds (such as NaH_2_PO_4_) may affect indicators of renal health in cats (Alexander et al 2018, Dobenecker et al 2018 a, b). More research is needed to clarify potential risk.”^92^

**TABLE 2:** Calcium and phosphorus recommendations for cats

	Calcium (minimum)		Phosphorus (minimum)		
	g/Mcal	g/100 g DM	g/Mcal	g/100 g DM	Ca : P ratio
FEDIAF					
Growth and reproduction	2.5	1.0	2.1	0.84	1 : 1 to 1.5 : 1
Adults, 100 kcal/kg^0.67^	1.48	0.59	1.25	0.50	1 : 1 to 2 : 1
Adults, 75 kcal/kg^0.67^	1.97	0.79	1.67	0.67	1 : 1 to 2 : 1
AAFCO					
Growth and reproduction	2.5	1.0	2.0	0.8	NR^b^
Adults	1.5	0.6	1.25	0.5	NR^b^
NRC (RA)^a^					
Growth and reproduction	2.7		1.9		NR^b^
Adults	0.72		0.64		NR^b^

^a^ RA, recommended allowance.

^b^ NR, no recommendation.

United States pet food regulations require all ingredients, including specific sources of P, to be listed individually in descending order by weight but this is not required for European pet foods. Furthermore, neither US nor European regulations require that total amounts of P or Ca be listed on commercial pet food labels. Many cat foods in the United Kingdom are not in compliance with published nutritional guidelines, and more relevant to the topic of this paper, about 10% have inverse Ca : P ratios (below 1 : 1).^93^ Likewise, among 82 cat foods sold in the United States, approximately 16% had an inverse Ca : P ratio.^94^ Given that the combination of low Ca : P ratios coupled with use of some highly soluble Pi salts are associated with markers of renal injury, it is important to address this potential danger. Although much remains to be studied, currently available data suggests the need to maintain dietary Ca : P at or above 1 : 1, while also avoiding excess inorganic Pi salts. However, specific guidelines regarding inorganic Pi salts require additional data as different inorganic Pi show differences in bioavailability and may differently impact renal health.^27^, ^45^, ^88^

## 6 WHAT CONCLUSIONS CAN AND CANNOT BE DRAWN BASED ON CURRENTLY AVAILABLE EVIDENCE?

Based on currently available evidence, it is clear that excess phosphorus, especially when provided as highly soluble inorganic phosphate salts, can be damaging to kidneys. This effect appears to be more pronounced in foods with an inverse Ca : P ratio. What is not known is the impact of various phosphate salts which vary in digestibility and physiological effects. Safe upper limits have not been established for the various Pi salts. Adverse effects can occur with high Pi diets in relatively short term studies, but might lower amounts be detrimental over longer periods or do physiological adaptations occur to protect against these effects and, if so, do individuals vary in their ability to adapt? Other dietary factors, such as the presence of phytates, fibers, other minerals, differences in base excess and differences in the base diet matrix, also may alter the impact of dietary P. No data currently shows that commercial diets induce renal injury. However, some commercial diets contain high amounts of phosphorus relative to recommendations and some have inverse Ca : P ratios, so could increase the risk for development of CKD.

It is clear that more research is needed. Until more evidence is available, the authors recommend avoiding diets with a Ca : P less than 1 : 1. Postprandial increases in serum Pi and PTH can occur when highly soluble sodium phosphates are added to diets in amounts equal to or greater than 0.5gP/Mcal, despite a Ca : P ratio above 1 : 1. However, similar results do not occur with other inorganic phosphates, such as calcium phosphates, and many inorganic phosphate sources have not yet been evaluated in this manner. While limiting the use of highly soluble phosphates, such as sodium‐ or potassium‐phosphates, appears to be important, there are insufficient data to support a specific upper limit for inorganic or soluble phosphates at this time. Among the additional research needed are studies to identify sensitive markers of renal injury; to identify risk, if any, from current commercial products; and to generate more information about the kinetics, impact of different phosphorus sources, and the physiological adaptations to long term feeding of highly bioavailable phosphates.

## CONFLICT OF INTEREST DECLARATION

Expenses related to the development of the manuscript (travel, meeting and honoraria) were offset by the sponsors, the Purina Institute and the Waltham Petcare Science Institute. Laflamme is a consultant for Purina. Backus receives support for his nutrition program from a Purina endowment, has consulted for both sponsors, and has received other support for his nutrition program from both sponsors and Hill's Pet Care. Butterwick and Czarnecki‐Maulden are employed by the sponsors. Elliott has served as a consultant for Waltham and has received research funding from Royal Canin. Fascetti serves on an advisory council and is a frequent speaker for the Purina Institute, and has received support for her nutrition program from both sponsors and Hill's Pet Care.

## OFF‐LABEL ANTIMICROBIAL DECLARATION

Authors declare no off‐label use of antimicrobials.

## INSTITUTIONAL ANIMAL CARE AND USE COMMITTEE (IACUC) OR OTHER APPROVAL DECLARATION

Authors declare no IACUC or other approval was needed.

## HUMAN ETHICS APPROVAL DECLARATION

Authors declare human ethics approval was not needed for this study.
